# Phosphodiesterase Inhibition Increases CREB Phosphorylation and Restores Orientation Selectivity in a Model of Fetal Alcohol Spectrum Disorders

**DOI:** 10.1371/journal.pone.0006643

**Published:** 2009-08-14

**Authors:** Thomas E. Krahe, Weili Wang, Alexandre E. Medina

**Affiliations:** Department of Anatomy and Neurobiology, Virginia Commonwealth University Medical Center, Richmond, Virginia, United States of America; Chiba University Center for Forensic Mental Health, Japan

## Abstract

**Background:**

Fetal alcohol spectrum disorders (FASD) are the leading cause of mental retardation in the western world and children with FASD present altered somatosensory, auditory and visual processing. There is growing evidence that some of these sensory processing problems may be related to altered cortical maps caused by impaired developmental neuronal plasticity.

**Methodology/Principal Findings:**

Here we show that the primary visual cortex of ferrets exposed to alcohol during the third trimester equivalent of human gestation have decreased CREB phosphorylation and poor orientation selectivity revealed by western blotting, optical imaging of intrinsic signals and single-unit extracellular recording techniques. Treating animals several days after the period of alcohol exposure with a phosphodiesterase type 1 inhibitor (Vinpocetine) increased CREB phosphorylation and restored orientation selectivity columns and neuronal orientation tuning.

**Conclusions/Significance:**

These findings suggest that CREB function is important for the maturation of orientation selectivity and that plasticity enhancement by vinpocetine may play a role in the treatment of sensory problems in FASD.

## Introduction

Fetal Alcohol Spectrum Disorders (FASD) is an umbrella term describing a range of effects that can occur in an individual whose mother imbibed alcohol during pregnancy. This condition is considered the leading cause of mental retardation in the western world with as many as 40,000 cases per year in the United States [1]. The sensory cortex is one of the most affected areas in FASD and children with this condition present altered somatosensory, auditory and visual processing [2]–[5], and often autistic behavior [6]. There is growing evidence indicating that these sensory problems may be related to poor cortical map refinement, organization and plasticity [7]–[11]. However, the mechanisms that underlie such effects remain to be elucidated.

The development of sensory cortical maps generally involves an initial phase in which the basic structure of the map is formed, followed by a refinement phase in which connections are eliminated and strengthened by activity-dependent mechanisms [12]–[14]. Several lines of research indicate that the later phase relies on a delicate balance between excitation and inhibition and the activation of plasticity-related genes [15]–[21]. Early alcohol exposure can disrupt this balance in many ways such as reducing and increasing NMDA and GABA receptors function respectively [22]–[26] and altering calcium storage and release [27], [28], which ultimately may affect gene expression [29]–[31]. The unspecific nature of the alcohol insult constrains the development of therapeutic approaches for sensory problems observed in models of early alcohol exposure. In fact, to date there is neither a cure nor an effective treatment for FASD. However, one possible approach might be to use pharmacological or molecular tools to improve the cellular machinery responsible for neuronal plasticity.

The cAMP response element-binding protein (CREB) is regulated by phosphorylation in response to neuronal activity patterns [32] and its role in neuronal plasticity has been long established. For instance, CREB function is required for consolidation of long-lasting plasticity, memory formation and circuitry development in several neuronal systems [19], [29], [33], [34]. Thus, the importance of CREB in regulating gene function and integrating physiological signals makes it an interesting molecular target to possibly overcome problems caused by early alcohol exposure. Therefore, intervening directly with CREB activation during development may ultimately allow for the maturation of sensory maps. Our recent findings [35] and other studies [36], [37] suggest that phosphodiesterase (PDE) inhibitors might be good candidates for enhancing CREB activation. PDE inhibitors prevent the breakdown of cAMP to 5′-AMP, prolonging the activation of protein kinases that promote phosphorylation of CREB [38], which in turn can enhance learning and memory in normal subjects [39] and restore ocular dominance plasticity in a FASD animal model [35].

Here we show that early alcohol-exposure leads to a persistent impairment in CREB phosphorylation and that treatment with a PDE type 1 inhibitor, several days after the period of the alcohol insult, restores normal phosphorylation of CREB, which in turn leads to normal development of orientation selectivity maps and single cell orientation tuning, cortical features known to be disrupted by early alcohol exposure [9], [40]. These findings show for the first time that phosphorylation of CREB is critical for the maturation of orientation selectivity and suggest that plasticity enhancement by pharmacological agents may play a role in the treatment of sensory problems in FASD.

## Results

In order to mimic alcohol binge drinking during the third trimester of pregnancy in humans, ferrets were injected with ethanol (3.5 g/kg, 25% in saline, i.p.) every other day between postnatal day (P) 10 to P30. This period is roughly equivalent to P4–P10 in rodents and to the third trimester equivalent of human gestation [9], [41], and encompasses the time when thalamic axons start connecting with layer IV neurons in the primary visual cortex [42]. Our previous findings revealed that the blood alcohol levels (∼250 mg/dl after 1–3 hours) as well as the frequency of alcohol exposure during this period disrupts orientation selectivity, without affecting visual responses [9]. Moreover, daily inspections of treated animals showed that alcohol exposure did not affect the time of eye opening (around P32 in the ferret).

Six days after the end of the alcohol treatment, animals received vinpocetine (40 mg/kg oral or 20 mg/kg i.p., see methods) or vehicle every day between P36 to P41, roughly the time of maturation of orientation selectivity in the ferret [43]. Vinpocetine treatment was done in a daily basis to maximize its effects, since our preliminary study done in rats indicated that the peak of vinpocetine action is within 8–12 hours after injection (supplemental material, Results S1, Figure S1). Optical imaging of intrinsic signals experiments were then conducted between P43 and P58 to examine the effects of vinpocetine treatment on the restoration of orientation selectivity.

### Restoration of orientation selectivity maps

**Figure 1:**
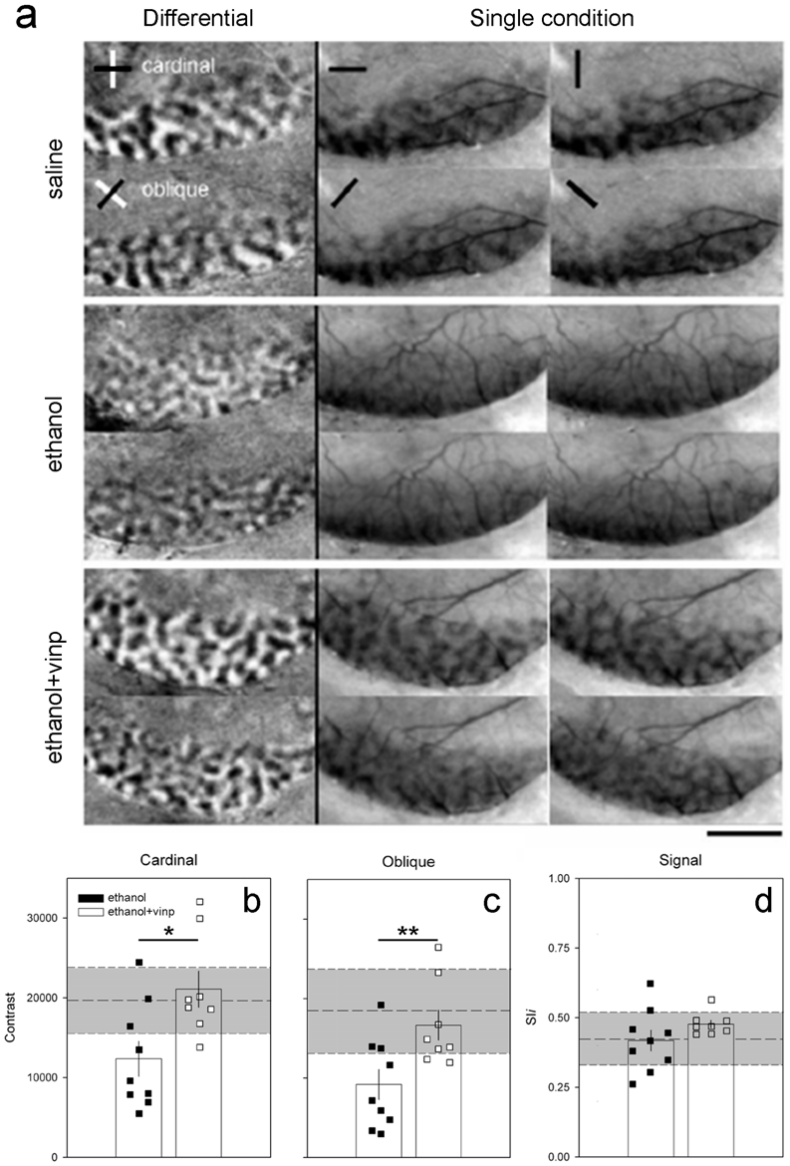
Restoration of contrast levels in orientation maps by vinpocetine treatment. (a) Orientation selectivity maps from representative animals of Saline, Ethanol and Ethanol+Vinpocetine groups, as revealed by optical imaging of intrinsic signals. Note that vinpocetine treatment restored the high contrast and modularity in differential and single condition maps respectively. Scale bar, 3 mm. (b and c) Quantification of contrast differences displayed for cardinal (b) and oblique (c) differential maps of Ethanol (n = 9 animals) and Ethanol+Vinpocetine (n = 9 animals) treated groups. Bars represent means (±SEM), and squares represent individual animal values. Dashed line represents the average contrast of Saline (n = 6 animals) treated animals and gray shaded area±SD. Note that most of the animals in the Ethanol group have a lower contrast than controls, while the great majority of animals in the Ethanol+Vinpocetine group present contrast values equal or similar to saline ones (Ethanol Vs Ethanol+Vinpocetine: * p = 0.025, cardinal and ** p = 0.002, oblique; Bonferroni). (d) Response to visual stimulation measured by pixel intensity plotted as a function of a signal intensity index (SI*i*, see methods). Note the similarity between groups.

Figure 1a shows representative cases confirming our previous findings that orientation maps in ethanol treated animals had markedly reduced contrast [9], so that the orientation domains were poorly defined at both cardinal and oblique orientations. The poor differential maps of Ethanol treated animals are unlikely to be explained by a peripheral effect of ethanol to the visual system [9], with Ethanol treated animals presenting visual acuity similar to controls (supplemental material, Results S1, Figure S2). Remarkably, Ethanol+Vinpocetine treated animals exhibited well defined and high-contrast differential maps with normal spacing and pattern of domains at both cardinal and oblique orientations, similar to maps observed in Saline treated animals (Figure 1a, differential maps). Moreover, single condition maps (Figure 1a) show that the reduction in contrast in Ethanol treated animals did not result from poor responses to visual stimulation. Ethanol treated animals showed strong cortical signals in response to visual stimulation at a single orientation, with the entire dorsal surface of V1/V2 uniformly darker than the background. Saline and Ethanol+Vinpocetine treated animals also showed strong cortical signals, but with a clear alternation of dark (visually responsive) and gray (non-responsive) areas (compare single condition maps in Figure 1a).

To quantify the effects of vinpocetine treatment, we computed the contrast of differential maps (see methods), as shown in Figure 1(b, and c). A value near 20,000 indicates a high contrast level (sharp orientation domains definition), and a value close to 10,000 indicates a low contrast level (poor orientation domains definition). Mean contrast values (±SEM) for cardinal and oblique orientations in Ethanol+Vinpocetine (cardinal: 21,083.99±2262.95; oblique: 16,650.97±1890.02; n = 8 ferrets) treated animals were similar to Saline (cardinal: 19,656.18±1246.99; oblique: 18,472.25±1586.58; n = 11) treated animals, and higher than in Ethanol (cardinal: 12,351.60±2193.15; oblique: 9,186.30±1887.37; n = 9) treated animals (Figure 1b and c). Oneway ANOVA (cardinal, F = 6.08, P = 0.007; oblique, F = 7.816, P = 0.002) followed by a Bonferroni test showed that Ethanol treated animals were significantly different from Saline (cardinal, p = 0.025; oblique, p = 0.002) and from Ethanol+Vinpocetine animals (cardinal, p = 0.012; oblique, p = 0.027). This analysis confirms our observations that Ethanol treated animals present poorly defined orientation domains [9] and shows that vinpocetine can restore its normal pattern. Quantification of signal intensity observed in the single condition maps (Figure 1d; see methods), revealed that the mean pixel intensity (±SEM) of Ethanol (0.42±0.04; n = 9 ferrets), Saline (0.42±0.03; n = 11 ferrets), and Ethanol+Vinpocetine (0.48±0.01; n = 8 ferrets) treated animals were similar (Oneway ANOVA, F = 1.62, P = 0.219), thus supporting that the response to visual stimulation in these animals was not reduced.

### Re-establishment of the orientation selectivity homogeneity

**Figure 2:**
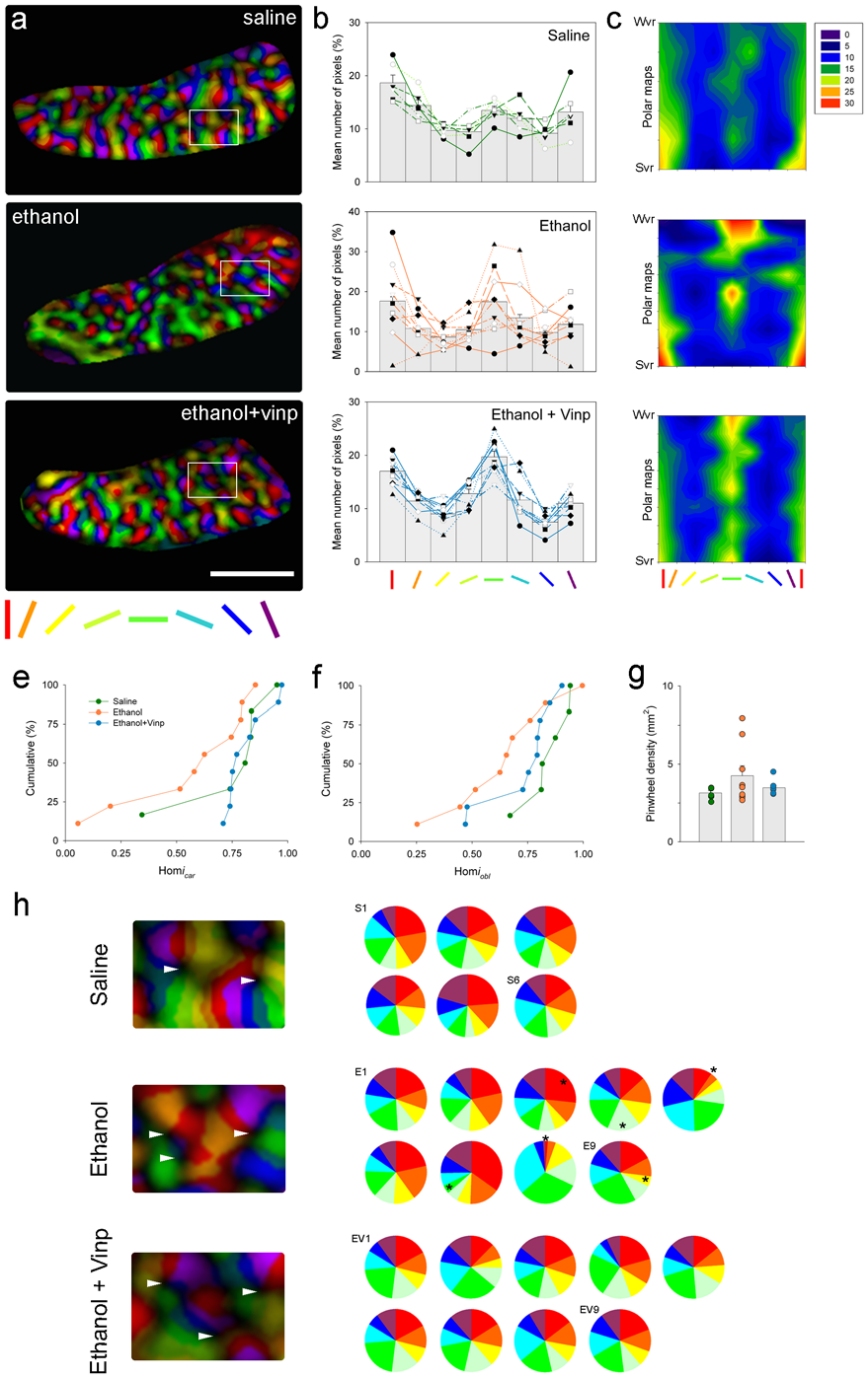
Re-establishment of orientation domains' homogeneity by vinpocetine treatment. (a) Polar magnitude maps of representative animals (left column), where brightness and colors represent signal strength and preference respectively. Note that both Saline and Ethanol+Vinpocetine animals present an even representation of colors. In contrast, the Ethanol animal presents an uneven representation of colors. Scale bar, 3 mm. (b) Normalized color distribution histograms (middle column) created from polar magnitude maps of Saline (n = 6), Ethanol (n = 9) and Ethanol+Vinpocetine (n = 9) treated animals. Bars represent means (±SEM), and symbols connected by colored lines represent individual animal values. (c) Contour plots of the color distribution data shown in b. Polar plots are sorted based on the visual response to 0° (cardinal) stimulation, from the strongest to the weakest visual response (Svr and Wvr, respectively). Note that there is great variation in response magnitudes and placement across the different polar maps in Ethanol treated animals. In contrast, Saline and Ethanol+Vinpocetine treated animals show a more homogeneous response profile across different polar maps with peak responses at 0° and 90° stimulus orientations. (e and f) Cumulative number of animals (in percent) plotted as a function of homogeneity indices (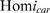
and 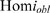, see methods) for all groups. Curves skewed to the right indicate similarity of orthogonal responses for cardinal and oblique orientations. (g) Quantification of pinwheel densities for Saline, Ethanol and Ethanol+Vinpocetine groups. Bars represent means (±SEM), and symbols individual animal values. (h) Representative pinwheel centers (arrow heads, left column) from polar maps shown in (a). Pie charts (right column) depict the amount of cortical responsiveness to different orientations related to the total number of pinwheel centers for Saline (S), Ethanol (E) and Ethanol+Vinpocetine (EV) treated animals. Proportions are based on the percentage of pixels of each pseudo-color in a particular polar map. Each chart represents a single animal (S_1–6_, E_1–9_, EV_1–9_). Note the uneven distribution of colors in most of alcohol exposed animals (indicated by *) and that Vinpocetine treated animals present color distributions similar to Saline treated animals.

Cortical responsiveness to visual stimulation at different angles is considerably homogeneous and has a tendency to be preferentially activated by vertical and horizontal contours than by contours at oblique angles (oblique effect [44]). Figure 2a illustrates polar plots of representative cases from Saline, Ethanol, and Ethanol+Vinpocetine treated groups. Ethanol treated animals presented an uneven representation of visual responses to different orientations whereas Saline and Ethanol+Vinpocetine treated animals showed a homogeneous representation of visual responses (seen as even color distributions) throughout V1 and V2. To quantify the contribution of visual responses to a particular orientation we computed the pseudo-color pixel distribution of polar maps from Saline, Ethanol, and Ethanol+Vinpocetine treated animals (Figure 2b and c, see methods). The majority of the Saline treated animals presented even color distributions with “peak” responses around the vertical and horizontal meridians (Figure 2b and c), indicating a tendency to have more visual cortical activation to contours in the cardinal axis compared with those at oblique angles. This result confirms previous findings in normal ferrets that slightly more of the primary visual cortex is devoted to processing cardinal than oblique orientations [44]. In contrast, Ethanol treated animals showed heterogeneous pixel distributions (Figure 2b and c) indicating that alcohol exposure during the development of orientation selectivity columns profoundly disrupts the intrinsic homogeneity and oblique effect observed in normal animals. Remarkably, Ethanol+Vinpocetine animals exhibited a striking recovery, with homogeneous pixel distributions peaking at the cardinal orientations similarly to Saline treated animals (Figure 2b and c).

The cumulative distributions of the Hom*i* values (see homogeneity index, methods) confirm that Vinpocetine treatment restores the intrinsic homogeneity in alcohol exposed animals (Figure 2e and f). Saline and Ethanol+Vinpocetine treated animals exhibited right-shifted curves (close to 1.0) when compared with Ethanol treated animals (Figure 2e and f), indicating that orthogonal responses for cardinal (0°+157 vs. 67.5°+90°) and oblique (22.5°+45° vs. 112.5°+135°) orientations present similar values (see methods). Kolmogorov-Smirnov comparisons revealed that the distribution of Hom*i* indexes in the Ethanol+Vinpocetine animals (n = 9) is similar to that of the Saline (n = 6, Z = 1.1, P = 0.16) and significantly different from that of the Ethanol treated animals (n = 9, Z = 2.1, P = 0.03).

### Pinwheel densities and relationship with orientation preference maps

A hallmark feature of the orientation selectivity cortical columnar organization is the presence of pinwheel centers: singularities in the orientation map around which all orientations are represented [45]. To further evaluate the effects of early alcohol exposure and vinpocetine treatment on cortical map organization, we counted the number of pinwheels (see methods) in Saline, Ethanol and Ethanol+Vinpocetine animals. Comparison of the number of pinwheel centers revealed that pinwheel densities were similar for all groups (Figure 2g) and similar to previous data in ferrets [46]. In Saline animals the mean pinwheel density (±SEM) was 3.14±0.15 (n = 6) per mm^2^ of cortical surface area; in Ethanol animals, there were 4.25±0.64 (n = 9) pinwheels/mm^2^; and 3.48±0.14 (n = 9) pinwheels/mm^2^ cortical surface in Ethanol+Vinpocetine treated animals. Figure 2h (left column) depicts pinwheel centers (arrow heads) of representative cases shown in Figure 2a. While pinwheel centers can be identified for all cases, only Saline and Ethanol+Vinpocetine present a more balanced distribution of visual responses surrounding these iso-orientation centers (Figure 2h).

### Restoration of orientation selectivity at the neuronal level

**Figure 3:**
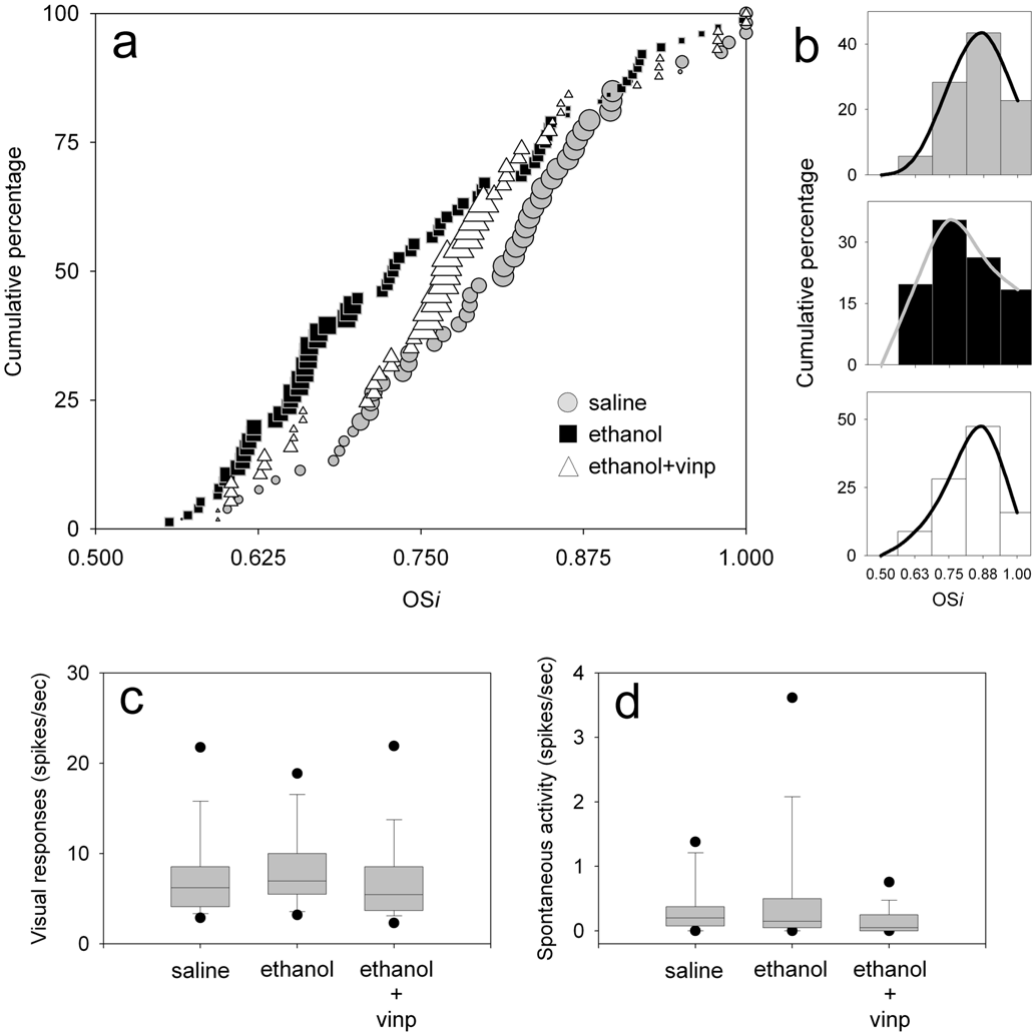
Vinpocetine promotes full recovery of cortical neuronal orientation selectivity and preserves normal visual responses to visual stimulation. (a) Cumulative percentage of cells is plotted as a function of an orientation selectivity index (OS*i*, see methods) for responses to the dominant eye in Saline (n = 53 cells), Ethanol (n = 76 cells) and Ethanol+Vinpocetine (n = 58 cells) treated animals. Symbol's size was based on the relative frequency of cells in 10 OS*i* intervals (0.51–0.55, 0.56–0.60, 0.61–0.65, 0.66–0.70, 0.71–0.75, 0.76–0.80, 0.81–0.85, 0.86–0.90, 0.91–0.95, and 0.96–1.00). (b) Histograms of OS*i* distributions shown in (a) for the three different groups. Note that in Ethanol treated animals, OS*i* distributions (in a and b) are skewed to the left compared to Saline and Ethanol+Vinpocetine distributions, indicating a greater proportion of poor selective cells. Distributions in (b) are based on the same cells in (a). (c) Maximum responses (in spikes per second), of the same neurons shown in (a), to visual stimulation at the optimal orientation for all groups. (d) Quantitative assessment of spontaneous activity for the same set of neurons. Box plots show the median, 10th, 25th, 75th, and 90th percentiles as vertical boxes with error bars. The fifth and 95th percentiles are shown as dots.

Although vinpocetine treatment restores orientation selectivity maps in alcohol exposed animals, we cannot discard the possibility that at the cellular level, neurons still present orientation tuning deficits [9]. To examine this possible scenario we conducted extracellular single-unit recordings in the binocular region of the primary visual cortex of alcohol exposed animals that received vinpocetine treatment. An orientation selectivity index (OS*i*) was obtained for each cell (see methods). An index of 1.0 indicates a high degree of selectivity. Figures 3a and b illustrate the OS*i* distributions for all groups. Ethanol treated animals' OS*i* distributions were significantly different from the distributions observed for Saline treated animals (Kolmogorov–Smirnov, Z = 1.57; P = 0.014). Similar to what was observed with optical imaging of intrinsic signals, vinpocetine treatment restored orientation selectivity in Ethanol treated animals (Saline vs. Ethanol+Vinpocetine, Z = 1.0, Kolmogorov–Smirnov, P = 0.262).

### Preservation of normal visual properties

We also made use of extracellular single-unit recordings to verify whether alcohol and vinpocetine treatments resulted in abnormal visually driven activity. Confirming our previous data [8], [9], [35], quantitative assessment of single-unit responses revealed similar mean maximal responses (in spikes per second) of cortical neurons to visual stimulation for all groups (Fig. 3c, F = 1.203, P = 0.30; univariate ANOVA). The mean maximal responses (±SEM) for the three groups were: Saline, 7.89±0.63 (53 cells), Ethanol 8.69±0.64 (76 cells), and Ethanol+Vinpocetine 7.19±0.67 (58 cells) respectively. Additionally, alcohol and vinpocetine treatments did not change spontaneous activity (recorded in the absence of visual stimulation) when compared to control levels (Figure 3d, Saline vs. Ethanol, P = 0.34; Saline vs. Ethanol+Vinpocetine, P = 0.40; Bonferroni). The mean spontaneous activity (±SEM) for the three groups were: Saline, 0.37±0.07 (53 cells), Ethanol 0.58±0.12 (76 cells), and Ethanol+Vinpocetine 0.16±0.03 (58 cells) respectively. However, alcohol exposed animals treated with vinpocetine presented a significant reduction in spontaneous activity when compared to animals that only received Ethanol treatement (Figure 3d, ANOVA, F = 5.3, P = 0.006; Bonferroni, P = 0.004). This result is in agreement with the observation that vinpocetine treated animals present sharper orientation selectivity maps (Figure 1 and 2) and more orientation selective cells (Figure 3a and b) than animals treated only with alcohol. In conclusion, the data shows that vinpocetine treatment after early alcohol exposure preserves robust responses to visual stimulation and normal signal to noise ratio in most cortical neurons.

### Restoration of pCREB levels

**Figure 4:**
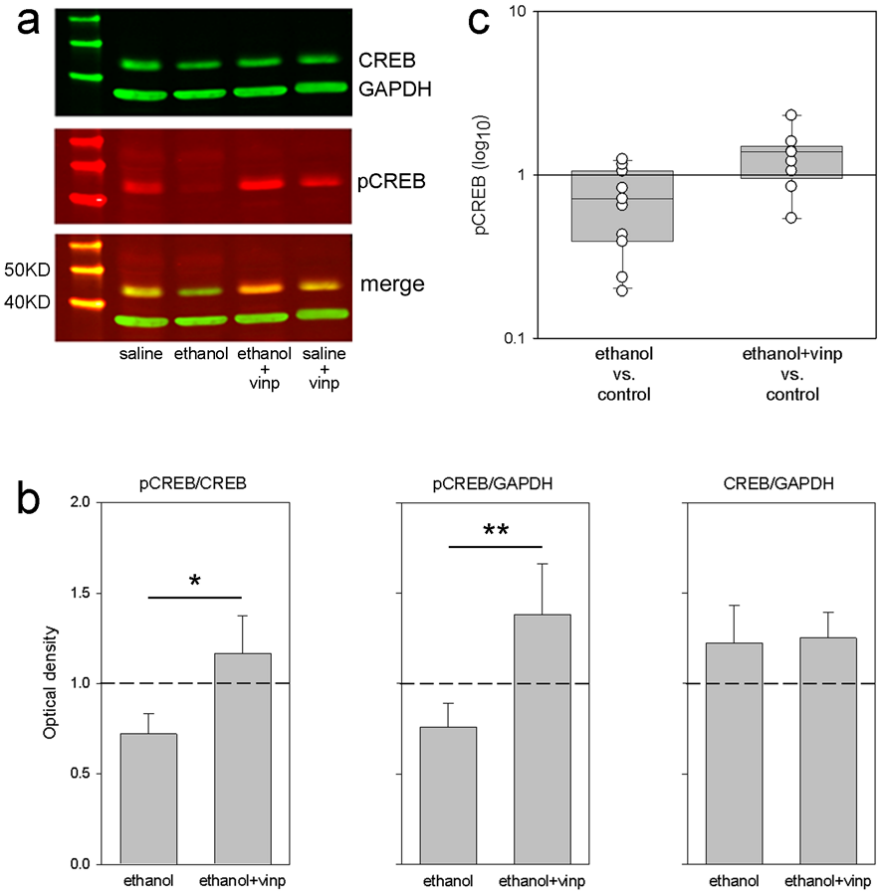
Vinpocetine treatment in alcohol exposed animals brings striate pCREB levels back to normal. (a) Representative fluorescent immunoblot showing pCREB (red) and CREB (green) levels. GAPDH (green) was used as loading control. Animals received ethanol or saline between P10–P30 and vinpocetine or vehicle at P36. Visual cortices were collected 9 hours (see supplemental material, Results S1, Figure S1) after vinpocetine administration. Note that pCREB levels were lower in Ethanol treated animals. PDE1 inhibition by vinpocetine restored pCREB to control levels. (b) Quantification of 5 immunoblots used to assess pCREB levels. Data normalized to controls. Average values for pCREB/CREB ratio as well as absolute pCREB levels (normalized by GAPDH) were higher in the Ethanol+Vinpocetine than in Ethanol animals (* P = 0.02 and ** P = 0.04, respectively; Paired t-test). Total CREB levels were similar in all groups. (c) Comparison of pCREB/GAPDH levels between groups. Each circle represents the value of a single animal normalized to its control in the same blot. Note that the majority of animals in the Ethanol and Ethanol+Vinpocetine groups present respectively lower and higher levels of pCREB when compared to controls.

To examine whether CREB function is related to the effects of early alcohol exposure on orientation selectivity as well as to its restoration by vinpocetine treatment we performed fluorescent immunoblots to assess pCREB levels in Saline (n = 10), Ethanol (n = 10) Ethanol+Vinpocetine (n = 8), and Saline+Vinpocetine (n = 10) animals. Similar to procedures done prior to physiological experiments, animals were exposed to ethanol or saline between P10–P30, and at P36 received a single dose of either vinpocetine (oral) or vehicle. We chose this age point since it is around the time that of orientation selectivity maturation in the ferret [43]. Nine hours after vinpocetine treatment, animals were euthanized with Euthasol (125 mg/kg, DelMarva labs, Midlothian) and their visual cortices collected. The timing for sample collection was based on a dose-response curve (supplemental material, Results S1, Figure S1) as well as previous studies [47]. The representative blot in Figure 4a indicates that pCREB levels were dramatically reduced in Ethanol treated animals but similar to control levels in Ethanol+Vinpocetine ones. Importantly, total CREB levels were neither affected by ethanol nor by vinpocetine. Quantification of immunoblots optical densities (Odyssey Application Software, 2.0) confirmed that vinpocetine treatment significantly increased CREB phosphorylation in Ethanol treated animals without affecting total CREB levels (Figure 4b). To further evaluate changes in CREB phosphorylation we compared pCREB/GAPDH levels of Ethanol and Ethanol+Vinpocetine animals to their controls (Figure 4c). To avoid the variability between blots we assured that each blot had at least one animal from each group, so that only bands in the same blot were directly compared. For the majority of Ethanol and Ethanol+Vinpocetine animals, pCREB levels were respectively lower and higher than control levels. Paired t-tests revealed significant differences between groups (Ethanol vs. Saline: t = 2.45, P = 0.037; Ethanol+Vinpocetine vs. Saline: t = 2.37, P = 0.049). These results indicate that: 1) vinpocetine treatment is effective in increasing the levels of pCREB in ethanol treated animals; 2) early alcohol exposure can promote long lasting impairment in CREB function; and 3) CREB function may be essential for cortical map development.

## Discussion

Here we show that pCREB levels remain decreased after the period of early alcohol exposure in a ferret model of FASD. This decrease could account for poor circuit refinement [48] and as a consequence, for the disrupted development of orientation selectivity shown in Ethanol treated animals [9]. Phosphodiesterase inhibition by vinpocetine, however, was able to restore pCREB levels in these animals, thus providing the appropriate conditions for circuitry refinement driven by visual experience, which in turn could account for the normal development of orientation selectivity.

### The importance of the CREB cascade for the development of orientation selectivity

The development of cortical orientation selectivity is known to require visual activity [14], [43], [49], [50]. However, how activity contributes to the development of cortical orientation selectivity or what the molecular basis for such activity-dependent processes is, still unclear. Orientation selectivity arises from a “feed-forward” process in which the spatial alignment of the on/off center receptive fields of geniculate afferents converge onto a simple cortical cell in layer IV. These simple cells in turn synapse onto layer II–III cells forming complex cells' receptive fields. Layer II–III cells are interconnected by horizontal connections, which link cortical columns that share iso-orientations domains together [13], [14], [51]–[53]. The establishment of this complex circuitry is believed to consist of an early experience-independent phase, in which the basic scaffold for cortical selectivity is achieved before eye opening, followed by a phase in which connections are refined by visual experience [13], [14], [50]. The prevailing theory used to explain the later experience-dependent plasticity phase relies on a Hebbian model in which mechanisms exist to strengthen synapses whose activity coincides with target depolarization beyond some threshold level [54] and to eliminate synapses whose activity is not correlated with postsynaptic activation [55]. This model requires a correlation detector that would signal synchronous pre- and post-synaptic depolarization. The characterization of NMDA voltage-dependent blockade mediated by Mg^2+^ [56]–[58] has led to the proposal that this subtype of glutamate receptor functions as a correlation detector [59]–[61], playing a critical role in activity-dependent synapse stabilization. Accordingly, previous studies in ferrets demonstrated that suppression of cortical NMDA function prevents the development of cortical orientation selectivity [62]. Further, inhibition has also been shown to play an important role in this model. It is believed that inhibitory circuits can “improve” the detection of activity imbalances by changing the cell's state relative to the threshold necessary for synapse stabilization [63]. Reduction of GABAergic inhibition after eye opening has been shown to perturb the development of cortical columnar architecture [64].

But how could visual activity result in the plastic changes necessary for circuit refinements? Visual stimulation activates the NMDA receptor allowing calcium influx that, in turn, activates protein kinases, such as Ca^++^/calmodulin-dependent kinases or cAMP-dependent protein kinase A. These kinases phosphorylate the transcription factor CREB [19], thereby activating it and leading to the transcription of plasticity-related genes that could ultimately strengthen synapses that exhibit high correlation or weaken/eliminate synapses that exhibit poor correlation. The importance of CREB function in the consolidation of long-term plasticity, memory formation and circuitry development has been demonstrated in several neuronal systems [19], [29], [33], [34]. In view of our present findings, the CREB cascade should also be considered an important factor in the development of cortical orientation selectivity.

### Effects of early alcohol exposure on the development of orientation selectivity

Here, animals were exposed to alcohol from the second to the fourth postnatal week (P10 to P30), a period when lateral geniculate neurons are forming synapses with layer IV neurons [42], and the orientation selectivity of cortical neurons start to develop [43], [49], [65]. Further maturation of orientation selectivity occurs after the end of alcohol exposure, until the adult state is reached around P38 [43], [49]. Vinpocetine treatment, on the other hand, was started on the sixth postnatal week (P36–P41), a period when the refinement of clustered horizontal connections takes place in the ferret [66], [67]. Based on this timeline, we can have a better understanding of how early alcohol exposure disrupts the development of orientation selectivity, and most importantly, how vinpocetine corrects it.

Alcohol may disrupt orientation selectivity by acutely suppressing NMDA receptor function [10], [23], [68] while enhancing GABA receptor function [69] thereby affecting CREB phosphorylation [70]–[72]. Following the end of ethanol exposure (P30), neocortical development and plasticity may be further disrupted as a result of substantial and long-lasting alterations of CREB activity [70]–[73], NMDA receptor activation [10], [26], and GABAergic mediated inhibition [24], [25], [74], [75]. These long-term disruptions during the development of the ferret's receptive field properties [76] may result in abnormal establishment of the neural circuits required for orientation selectivity.

The fact that vinpocetine treatment was able to restore orientation selectivity in animals early exposed to alcohol suggests that, although NMDA and GABA receptor functions are affected by alcohol [10], [24], [26], [68], [74], [75], the long-lasting alterations in CREB activity seem to be the main cause for the poor development of cortical orientation selectivity. When pCREB levels of alcohol exposed animals were restored during vinpocetine treatment, concomitantly visual activity triggered cortical cells and this activation was able to be translated into plastic changes that resulted in the normal refinement of cortical maps. In other words, NMDA and GABA receptors must be functional at some level, since systemic administration of vinpocetine resulted in the restoration of pCREB to cortical cells indiscriminately, yielding the recovery of cortical orientation selectivity. If this was not the case, the cellular “input” (NMDA and GABA receptors) would be unable to properly make the transduction of the visual information. Our extracellular single-unit results corroborate the idea that upstream factors in the cellular machinery are not severely affected in our model since alcohol treated animals present normal cellular response properties to visual stimulation (Figure 3c and d) and normal visual acuity (supplemental material, Results S1, Figure S2).

Another important aspect of our findings is that vinpocetine treatment takes place during the period of the refinement of layer II–III cortical horizontal connections in the ferret [66], [67]. Therefore, this suggests that CREB activity can play an important role in the strengthening and elimination of these connections. Accordingly, vinpocetine treated animals presented sharper orientation selectivity maps than Saline treated animals (Figures 1 and 2). However, our extracellular recordings indicate that at the neuronal level, Saline treated animals presented more orientation selective cells than Ethanol+Vipocetine treated animals did (Figures 3a), although this difference did not reach statistical significance. Taken together these results reinforce the hypothesis that vinpocetine treatment in alcohol exposed animals may improve the refinement of horizontal connections since optical imaging is limited to signal acquisition from superficial cortical layers. On the other hand, single-unit data includes recordings not only from layers II–III but also from deeper layers. Previous studies in mice demonstrated the involvement of CREB in the refinement of retinogeniculate axons in the dorsal thalamus [48]. Our findings suggest that similar mechanisms may also take place in the visual cortex of higher mammals.

However, one may argue that vinpocetine's restorative effects on orientation selectivity maps might be in part explained by its cerebral vasodilatory properties. While vinpocetine has vasodilatory effects [77] and optical imaging of intrinsic signals relies on changes in light refraction caused by hemodynamic signals [78], it is unlikely that this would explain the restoration of orientation selectivity maps seen in our study. Previous findings demonstrated that optical imaging of intrinsic signals is related to blood oxygenation changes rather than increases in blood flow, which yields its great spatial resolution [78]–[81] . While the effect of oxygen consumption by active cells is localized to a cortical column, the compensatory blood inflow seems to be spread out over multiple columns [78]–[81]. Moreover, at the time of optical imaging experiments the animals were no longer under the effects of vinpocetine and the single unit and optical imaging data showed similar results.

### Clinical relevance

Orientation selectivity is a property of visual cortex neurons that is thought to be essential for visual perception of shapes and borders [13], [14], [44], [82]. While there are no studies showing whether children with FASD have poor orientation selectivity maps, neuropsychological findings showing that patients with FASD present poor performance in perception of geometric designs support this hypothesis [3], [4]. Our present findings indicate that orientation selectivity maps once disrupted by early alcohol exposure can be restored by vinpocetine treatment. Therefore, our results suggest that PDE1 inhibitors could be used to ameliorate sensory deficits presented in children with FASD. Moreover, given that orientation selectivity circuitry maturation mechanisms and organization are likely to be present in other areas of the neocortex [13], [83], [84], it is plausible to consider that PDE1 inhibitors may not be applicable exclusively to FASD.

### Conclusion

In conclusion, we showed that administration of a PDE1 inhibitor several days after the period of alcohol exposure can improve cortical organization in the ferret model of FASD by restoring pCREB levels. Improving activity-dependent plasticity using PDE1 inhibitors should be seriously considered in other animal models of impaired cortical development, since orientation selectivity map formation is believed to share similarities to the development of other cortical areas [13], [83], [84], and because impairments in sensory function are present in many other neurodevelopmental disorders [85]–[89].

It is of great importance to devise treatment approaches that can be implemented long after the period of alcohol exposure. However, the biggest challenge to this has been the non-specific nature of the alcohol insult. Early alcohol exposure affects many neuronal biochemical and physiological properties [26]. To restore each one of these deficits is an unrealistic endeavor. However, it may be possible to ameliorate some of the neurobehavioral problems observed in FASD by using PDE inhibitors to enhance neuronal plasticity and improve sensory function [90]. In this regard, our findings could be of extreme importance from a clinical standpoint since they open the possibility for treating juveniles when prevention fails.

## Methods

All procedures described in this paper were approved by the Institutional Animal Care and Use Committee at Virginia Commonwealth University.

### Treatments

Ferrets were treated with 3.5 g/kg alcohol i.p. (25% in saline) or saline as control every other day between postnatal day (P) 10 to P30. This alcohol treatment leads to a blood alcohol level of approximately 250 mg/dl for 1–5 hours after injection [8]. Between P36–P41 Ethanol treated animals received either 40 mg/kg of vinpocetine (oral, mixed in Nutrical©, n = 6) or 20 mg/kg (i.p., dissolved in DMSO, n = 3). The results were similar between these groups and the animals were pooled together. As a control, alcohol treated animals received Nutrical© (oral, n = 7) or DMSO solution (i.p., n = 2). The results were also similar between these two control groups and these animals were pooled together. Integrity of orientation selectivity columns were evaluated 1–7 days after vinpocetine (or control) treatment by optical imaging of intrinsic signals. Orientation tuning at the neuronal level was analyzed by single-unit recordings (Saline n = 4; Ethanol n = 5 and Ethanol+vinpocetine n = 6). Levels of pCREB and CREB were obtained in immunoblots from Saline (n = 10), Ethanol (n = 10) and Ethanol+Vinpocetine (n = 8) animals. Similar to the procedures done prior to physiological experiments, animals were exposed to ethanol or saline between P10–P30, and at P36 received a single dose of either vinpocetine (oral) or vehicle. Nine hours later, animals were euthanized with Euthasol (125 mg/kg, DelMarva labs, Midlothian, VA) and their visual cortices collected.

### Optical imaging of intrinsic signals

Animals were premedicated by subcutaneous injection of a tranquilizer (acepromazine, 1 mg/kg), and a muscarinic antagonist (methyl atropine bromide, 0.2 mg/kg) to reduce bronchial secretion, as well as dexamethasone sodium phosphate (0.5 mg/kg) to reduce inflammation. Animals were then anesthetized using sodium pentobarbital (35 mg/kg, Abbott Laboratories, North Chicago, IL) and placed in a stereotaxic frame. No procedures were started until the animal was sufficiently anesthetized, as ascertained by the loss of withdrawal and cornea-blink reflexes. A tracheal cannulation was performed, and the animal was placed on a ventilator and paralyzed using pancuronium bromide (0.2 mg/kg, i.p.). To comply with NIH guidelines for use of paralytic agents and to certify that the animals were maintained at an appropriate level of anesthesia, use of muscle relaxants was omitted in some experiments and withdrawal reflexes were monitored in these animals. Similar procedures have been previously described and shown to be appropriate for visual physiology studies conducted in ferrets [8], [9], [35], [91]. Heart rate, expired CO_2_ and arterial blood oxygen saturation (SpO_2_) were monitored continuously and maintained at approximately 270 bpm, 4.0% and above 90%, respectively. Body temperature was maintained at 38°C using a homeostatic blanket. Supplemental doses of pentobarbital (12 mg/kg) were given every hour throughout the experiment or when heart rate or expired CO_2_ increased, a procedure previously shown to preserve visual responses over time [8]. Nictitating membranes were retracted using phenylephrine hydrochloride (2.5%), the pupils were dilated with atropine sulfate (1%) and contact lenses were placed on the corneas. Subcutaneous injections of 10% dextrose and 0.9% saline were given to prevent dehydration. Clarity of optical media and retina integrity was evaluated before and during each experiment with the aid of an ophtalmoscope. No animal presented opacity or any evident alteration that would affect proper vision.

Optical imaging of intrinsic signals was performed with Imager 2001 VSD+ (Optical Imaging System Inc., Germantown, NY) using imaging methods slightly modified from those described elsewhere [8], [9], [35], [91]. A craniotomy was made over the left hemisphere to expose the dorsal area of the occipital cortex. The dura was reflected and the opening filled with agar (2.5% in saline) and covered with a glass coverslip. An image of the vascular pattern was then obtained by illuminating the cortical surface with a green filter (approximately 550 nm) using a tungsten-halogen light source. Next, images of intrinsic signals were obtained using a red filter (approximately 700 nm). Visual stimulation consisting of high-contrast square wave gratings (8.75° dark phase/1.25° light phase) was generated on a 21-inch monitor (Sony Trinitron) using SGT+ graphics board and STIM software. Gratings were presented to both eyes at an angle of 0°, 45°, 90° or 135° and drifted (22.5°/sec) in both directions along the axis orthogonal to the orientation of the grating. A single trial consisted of these four gratings and a blank screen presented to each eye for 9 sec in a pseudorandom sequence, with data acquisition during the last 8 sec. A total of 20 trials were performed for each eye and the summed images were used to obtain single condition maps by subtracting responses to each angle (0°, 45°, 90° and 135°) from responses to a blank screen. In these images, dark areas correspond to regions responsive to a specific angle. In addition, differential maps (cardinal and oblique) were obtained by subtracting the optical signal at one orientation from the response obtained at the orthogonal orientation (cardinal: 0°–90°; oblique: 45°–135°). For some animals, four intermediate angles (22.5°, 67.5°, 112.5°, and 157.5°) were also employed to generate polar maps.

### Differential maps–contrast analysis

Quantitative analysis of orientation selectivity was done by assessing the contrast level of the differential maps. To obtain a quantitative estimate of contrast texture, differential maps were mixed with the floating point files clipped at ±3SD from the median. The resulting 8 bit difference images were rescaled from 0 to 255 and the contrast was calculated based on the Grey-Level-Co-Occurrence Matrix contrast texture technique described by Haralick and colleagues [92]. This analysis quantifies the dependencies between neighboring pixels and patterns of variation in image brightness within a region of interest (ROI). We used Image J's (version 1.37, National Institutes of Health, USA) Texture Analyzer plug-in (Julio E. Cabrera, http://rsbweb.nih.gov/ij/plugins/texture.html) to compute the contrast texture of differential maps (cardinal and oblique). Three contrast values were obtained for each differential image (248×160 pixels) from 3 non-overlapping ROIs (55×55 pixels) that included V1 and V2, covering nearly the entire map surface. The contrast values were then summed to obtain a contrast value for a single differential map. A value near to 20,000 indicates a high contrast level (high degree of selectivity), and a value close to 10,000 indicates a low contrast level (poor degree of selectivity).

### Differential maps–signal strength

To compare the strength of visual responses across the different treatment groups we computed the signal intensity observed in single condition maps. An estimate of signal intensity was obtained by first computing the pixel distribution along a grayscale containing 128 levels of gray in 8 bit single condition images clipped at ±3SD from the median. In this scale, 0 represents lack of responses and 127 the strongest possible response to visual stimulation. For each single condition map (0°/blank stimulus [bs], 45°/bs, 90°/bs and 135°/bs), the pixel distribution was obtained from an ROI drawn manually to include V1 and V2. To avoid sampling biases, for each animal, ROIs were outlined on maps of total visual response (0°+45°+90°+135°/blank stimulus). Next, a signal intensity index (SI*i*) was calculated for each condition according to the following formula:



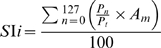
where P is the number of pixels in each one of the 128 grayscale levels, P*_t_* is the total number of pixels in the ROI and A*_m_* is an arbitrary number ranging from 1.0 to 100.0 such that for each grayscale level there is a correspondent A*_m_* value. Thus, an SI*i* value of 1.0 would indicate that the entire visual cortex is maximally activated. However, signal intensity is expect to vary within a single condition map [9], reflecting alternation of regions with high signal intensity located within the orientation columns and regions with low signal intensity located outside of the columns. For each animal, the SI*i* for each one of the 4 single conditions was averaged.

### Homogeneity index

Cortical responsiveness to visual stimulation at different angles is considerably homogeneous and preferentially activated by cardinal contours rather than by contours at oblique angles (oblique effect [44]), with response peaks around 0° and 90° orientations. To verify this intrinsic property of the visual system of higher mammals we first calculated the pixel distribution of pseudo-colors in polar maps for all treated groups. Briefly, the 8 bit images were clipped at ±3SD from the median and color histograms were made using Image J software (Color Histogram plug-in, Kai Uwe Barthel, Internationale Medieninformatik, Berlin, Germany, http://rsbweb.nih.gov/ij/plugins/color-inspector.html) in an ROI drawn manually to include V1 and V2. To avoid biases in the identification of the area of cortical responsiveness, ROIs were outlined on maps of total visual response (0°+45°+90°+135°/blank stimulus) and then superimposed on the respective polar map. Next, homogeneity indexes (Hom*i*) were computed from the pixel distributions according to the following formulas:
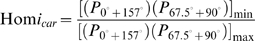


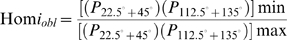



Where *P* is the sum of pixels from adjacent stimuli orientations, and 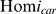
and 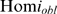 correspond to responses from cardinal and oblique stimulus orientations respectively. The response to each one of the eight angles (number of pixels) was normalized for all treated groups. Indices of 1.0 indicate similar responses across orthogonal orientations.

### Pinwheel counting

Pinwheel numbers and densities were determined in a similar way as previously described elsewhere [93]. Briefly, identification of pinwheel centers was based on the crossing of the 0°/90° with the 45°/135°-orientation contours, respectively. Pinwheel centers were visually counted with the aid of Image J software (version 1.37, National Institutes of Health, USA) from an iso-orientation contour image derived from the corresponding angle map. To avoid any counting bias, the experimenter was unaware of the treatment condition of the animals. The angle map area was calculated from an ROI drawn manually to include V1 and V2 and then superimposed on the respective polar map. To avoid biases in identification of the area of cortical responsiveness, ROIs were outlined on maps of total visual response (0°+45°+90°+135°/blank stimulus). The total number of pixels was sampled using Image J. The average (±SEM) area sizes of cortical maps were similar between all groups: 268.36±12.02 mm^2^ for Saline (n = 6 animals), 205.84±19.68 mm^2^ for Ethanol (n = 9 animals), and 253.21±9.26 mm^2^ for Ethanol+Vinpocetine (n = 9 animals). No significant size differences were observed (P = 0.088, Multivariate ANOVA).

We also investigated the relationship between the number of pinwheels and the cortical representation of visual responses to gratings of different orientations. For each polar map, we divided the number of pixels in each pseudo-color (see above) by the total number of pinwheels.

### In vivo electrophysiology

Animals were premedicated, anesthetized and ventilated with similar procedures described for optical imaging. Temperature, heart rate, expired CO_2_ and SpO_2_ were monitored continuously. A craniotomy (3–4 mm in diameter) was performed to expose the binocular region of the left primary visual cortex where recordings were performed. Single-unit recordings were conducted using a glass-coated tungsten microelectrode with a 5 µm exposed tip lowered into the primary visual cortex at approximately 20° to the vertical. To minimize sampling bias, single-units used in this study were separated by at least 100 µm along the electrode track. After the isolation of a single-unit, its receptive field was mapped and the optimal stimulus orientation, direction and velocity were determined qualitatively using a moving bar of light projected onto a tangent screen. Ocular dominance, spontaneous activity and number of spikes per stimulus were then quantitatively determined for each cell by presenting a computer-controlled bar of light to each eye. Each stimulus presentation consisted of the bar of light moving across the receptive field at the optimal orientation in one direction and back across in the opposite direction. To assess orientation selectivity, the moving bar of light was presented to each eye separately at four orientations centered around the optimal (0°, 45°, 90° and 135°). Spikes were collected during the 10 stimulus presentations by a computer using Spike 2 software (Cambridge Electronics Design, Cambridge, UK) and peristimulus histograms were generated. Spontaneous activity was determined by recording activity in the absence of stimulation. At the conclusion of the electrophysiology experiment, ferrets were killed with Euthasol, (125 mg/Kg, i.p.; Delmarva labs, Midlothian, VA).

### Neuronal orientation selectivity

To quantify the orientation selectivity based on the single-unit recordings, an orientation selectivity index (OS*i*) was obtained for each cell by using the following formula:
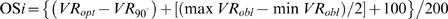



Where *VR* is the visual response (in spikes per second) to different orientations (*opt*, optimal; 90° to optimal; *obl*, 45° to optimal or 135° to optimal). Indices of 1.0 indicate a high degree of selectivity.

### Western blotting

Ferret visual cortices were dissected at P36 (n = 38), frozen on dry ice and stored at −80°C then homogenized in Tissue Extraction Buffer (Biosource, Carlsbad, CA) with a Protease and a Phosphatase inhibitor cocktail (Sigma, St. Louis, MO). Due to the fact that western blot studies normally require large sample sizes and that ferrets are a USDA protected species, we limited our western blot analysis to a single age point. Protein concentrations were measured using Bradford protein assay with bovine serum albumin as a standard (Bio-Rad, Hercules, CA). Next, 50 µg of total protein was resolved by SDS-PAGE (10% Tris-HCl Rgels, Bio-Rad, Hercules, CA) and transferred to nitrocellulose membranes (Bio-Rad, Hercules, CA). Blots were incubated in blocking buffer (LI-COR, Lincoln, NE) for 1 hour, then incubated in Phospho-CREB (Ser133) (87G3) rabbit mAb (1∶200, Cell Signalling Tech.) overnight at 4°C. This was followed by two hours of incubation in CREB (86B10) mouse mAb (1∶200, Cell Signalling Tech.) at room temperature, then one hour in GAPDH mouse mAb (Sigma, St. Louis, MO). The secondary antibodies were goat anti-mouse IRDye 800CW IgG and goat anti-rabbit IRDye 680 IgG (1∶4000, LI-COR, Lincoln, NE). Blots were detected by Odyssey Image System (LI-COR, Lincoln, NE).

## Supporting Information

Results S1 a)Effects of PDE type 1 inhibition on cAMP and pCREB levels. b)Alcohol exposure during the third trimester equivalent of human gestation does not affect visual acuity in the ferret.
(0.04 MB DOC)

Figure S1 Changes in cAMP and CREB phosphorylation after vinpocetine. cAMP levels assessed by a commercially available immunoassay kit. CREB phosphorylation assessed by western blotting. Primary antibody: pCREB (Cell signaling, 1∶200 dilution). pCREB data was normalized by actin.
(0.32 MB TIF)

Figure S2 Number of spikes resulting from stimulation with different spatial frequencies. While the ethanol treated animal presented a severe impairment of the orientation selectivity map its response to changes in spatial frequency was similar to a control animal that exhibited a highly organized orientation selectivity map.
(0.50 MB TIF)

## References

[1] Klug MG, Burde L (2003). Fetal Alcohol Syndrome prevention: annual and cumulative cost savings.. Neurotoxicol Teratol.

[2] Rasmussen C, Horne K, Witol A (2006). Neurobehavioral functioning in children with fetal alcohol spectrum disorder.. Child Neuropsychol.

[3] Mattson SN, Gramling L, Delis DC, Jones KL, Riley EP (1996). Global-local processing in children prenatally exposed to alcohol.. Child Neuropsychol.

[4] Uecker A, Nadel L (1996). Spatial locations gone awry: Object and spatial memory deficits in children with fetal alcohol syndrome.. Neuropsychologia.

[5] Coles CD, Platzman KA, Lynch ME, Freides D (2002). Auditory and visual sustained attention in adolescents prenatally exposed to alcohol.. Alcohol Clin Exp Res.

[6] Harris SR, MacKay LL, Osborn JA (1995). Autistic behaviors in offspring of mothers abusing alcohol and other drugs: a series of case reports.. Alcohol Clin Exp Res.

[7] Margret CP, Chappell TD, Li CX, Jan TA, Matta SG (2006). Prenatal alcohol exposure (PAE) reduces the size of the forepaw representation in forepaw barrel subfield (FBS) cortex in neonatal rats: relationship between periphery and central representation.. Exp Brain Res.

[8] Medina AE, Krahe TE, Coppola DM, Ramoa AS (2003). Neonatal alcohol exposure induces long-lasting impairment of visual cortical plasticity in ferrets.. J Neurosci.

[9] Medina AE, Krahe TE, Ramoa AS (2005). Early Alcohol Exposure Induces Persistent Alteration of Cortical Columnar Organization and Reduced Orientation Selectivity in the Visual Cortex.. J Neurophysiol.

[10] Rema V, Ebner FF (1999). Effect of enriched environment rearing on impairments in cortical excitability and plasticity after prenatal alcohol exposure.. J Neurosci.

[11] Powrozec TA, Zhou FC (2005). effects of prenatal alcohol exposure on the development of the vibrissal somatosensory cortical barrel network.. Brain Res Dev Brain Res.

[12] Katz LC, Shatz CJ (1996). Synaptic activity and the constuction of cortical circuits.. Science.

[13] Huberman AD, Feller MB, Chapman B (2008). Mechanisms underlying development of visual maps and receptive fields.. Annu Rev Neurosci.

[14] White LE, Fitzpatrick D (2007). Vision and Cortical map development.. Neuron.

[15] Nicoll R (2003). Expression mechanisms underlying long-term potentiation: a postsynaptic view.. Philos Trans R Soc Lond B Biol Sci.

[16] Mower AF, Liao DS, Nestler EJ, Neve RL, Ramoa AS (2002). cAMP/Ca2+ response element-binding protein function is essential for ocular dominance plasticity.. J Neurosci.

[17] Kleinschmidt A, Bear MF, Singer W (1987). Blockade of “NMDA” receptors disrupts experience-dependent plasticity of kitten striate cortex.. Science.

[18] Hensch TK, Fagiolini M, Mataga N, Stryker MP, Baekkeskov S (1998). Local GABA circuit control of experience-dependent plasticity in developing visual cortex.. Science.

[19] Frank DA, Greenberg ME (1994). CREB: a mediator of long-term memory from mollusks to mammals.. Cell.

[20] Fagiolini M, Fritschy JM, Low K, Mohler H, Rudolph U (2004). Specific GABAA circuits for visual cortical plasticity.. Science.

[21] Rao VR, Finkbeiner S (2007). NMDA and AMPA receptors: old channels, new tricks.. Trends Neurosci.

[22] Miller MW (2006). Effect of prenatal exposure to ethanol on glutamate and GABA immunoreactivity in macaque somatosensory and motor cortices: critical timing of exposure.. Neuroscience.

[23] Lovinger DM, White G, Weight FF (1989). Ethanol inhibits NMDA-activated ion current in hippocampal neurons.. Science.

[24] Hsiao SH, West JR, Mahoney JC, Frye GD (1999). Postnatal ethanol exposure blunts upregulation of GABAA receptor currents in Purkinje neurons.. Brain Res.

[25] Durand D, Carlen PL (1984). Decreased neuronal inhibition in vitro after long-term administration of ethanol.. Science.

[26] Costa ET, Savage DD, Valenzuela CF (2000). A review of the effects of prenatal or early postnatal ethanol exposure on brain ligand-gated ion channels.. Alcohol Clin Exp Res.

[27] Lee YH, Spuhler-Phillips K, Randall PK, Leslie SW (1996). Effects of prenatal ethanol exposure on voltage-dependent calcium entry into neonatal whole brain-dissociated neurons.. Alcohol Clin Exp Res.

[28] Servais L, Hourez R, Bearzatto B, Gall D, Schiffmann SN (2007). Purkinje cell dysfunction and alteration of long-term synaptic plasticity in fetal alcohol syndrome.. Proc Natl Acad Sci U S A.

[29] Bito H, Deisseroth K, Tsien RW (1996). CREB phosphorylation and dephosphorylation: A Ca++ and stimulus duration dependent switch for hippocampal gene expression.. Cell.

[30] Kornhauser JM, Cowan CW, Shaywitz AJ, Dolmetsch RE, Griffith EC (2002). CREB transcriptional activity in neurons is regulated by multiple, calcium-specific phosphorylation events.. Neuron.

[31] Lamprecht R (2005). CREB: a message to remember.. Cell Mol Life Sci.

[32] Deisseroth K, Tsien RW (2002). Dynamic multiphosphorylation passwords for activity-dependent gene expression.. Neuron.

[33] Deisseroth K, Bito H, Tsien RW (1996). Signaling from synapse to nucleus: postsynaptic CREB phosphorylation during multiple forms of hippocampal synaptic plasticity.. Neuron.

[34] Finkbeiner S, Tavazoie SF, Maloratsky A, Jacobs KM, Harris KM (1997). CREB: a major mediator of neuronal neurotrophin responses.. Neuron.

[35] Medina AE, Krahe TE, Ramoa AS (2006). Restoration of Neuronal Plasticity by a Phosphodiesterase Type 1 Inhibitor in a Model of Fetal Alcohol Exposure.. J Neurosci.

[36] Barad M, Bourtchouladze R, Winder DG, Golan H, Kandel E (1998). Rolipram, a type IV-specific phophodiesterase inhibitor, facilitates the establishment of long-lasting long-term potentiation and improves memory.. Proc Natl Acad Sci U S A.

[37] Monti B, Berteotti C, Contestabile A (2006). Subchronic rolipram delivery activates hippocampal CREB and arc, enhances retention and slows down extinction of conditioned fear.. Neuropsychopharmacology.

[38] Bender AT, Beavo JA (2006). Cyclic nucleotide phosphodiesterases: molecular regulation to clinical use.. Pharmacol Rev.

[39] Blokland A, Schreiber R, Prickaerts J (2006). Improving memory: a role for phosphodiesterases.. Curr Pharm Des.

[40] Medina AE, Krahe TE (2008). Neocortical plasticity deficits in fetal alcohol spectrum disorders: Lessons from Barrel and Visual Cortex.. J Neurosci Res.

[41] Clancy B, Darlington RB, Finlay BL (2001). Translating developmental time across mammalian species.. Neuroscience.

[42] Herrmann K, Antonini A, Shatz CJ (1994). Ultrastructural evidence for synaptic interactions between thalamocortical axons and subplate neurons.. Eur J Neurosci.

[43] Chapman B, Stryker MP, Bonhoeffer T (1996). Development of orientation preference maps in ferret primary visual cortex.. J Neurosci.

[44] Coppola DM, White LE, Fitzpatrick D, Purves D (1998). Unequal representation of cardinal and oblique contours in ferret visual cortex.. Proc Natl Acad Sci U S A.

[45] Bonhoeffer T, Grinvald A (1991). Iso-orientation domains in cat visual cortex are arranged in pinwheel-like patterns.. Nature.

[46] Rao SC, Toth LJ, Sur M (1997). Optically imaged maps of orientation preference in primary visual cortex of cats and ferrets.. J Comp Neurol.

[47] Miskolczi P, Korma K, Polgar M, Vereczkey L (1990). Pharmacokinetics of vinpocetine and its main metabolite apovincaminic acid before and after the chronic oral administration of vinpocetine to humans.. Eur J Drug Metab Pharmacokinet.

[48] Pham TA, Rubenstein JLR, Silva AJ, Storm DR, Stryker MP (2001). The CRE/CREB pathway is transiently expressed in thalamic circuit development and contributes to refinement of retinogeniculate axons.. Neuron.

[49] Chapman B, Stryker MP (1993). Development of orientation selectivity in ferret visual cortex and effects of deprivation.. J Neurosci.

[50] White LE, Coppola DM, Fitzpatrick D (2001). The contribution of sensory experience to the maturation of orientation selectivity in ferret visual cortex.. Nature.

[51] Hubel DH, Wiesel TN (1959). Receptive fields of single neurones in the cat's striate cortex.. J Physiol.

[52] Hubel DH, Wiesel TN (1962). Receptive fields, binocular interaction and functional architecture in the cat's visual cortex.. J Physiol.

[53] Hubel DH, Wiesel TN (1963). Shape and arrangement of columns in cat's striate cortex.. J Physiol.

[54] Hebb DO (1949). The organization of behavior..

[55] Stent GS (1973). A physiological mechanism for Hebb's postulate of learning.. Proc Natl Acad Sci U S A.

[56] MacDermott AB, Mayer ML, Westbrook GL, Smith SJ, Barker JL (1986). NMDA-receptor activation increases cytoplasmic calcium concentration in cultured spinal cord neurones.. Nature.

[57] Mayer ML, Westbrook GL, Guthrie PB (1984). Voltage-dependent block by Mg2+ of NMDA responses in spinal cord neurones.. Nature.

[58] Nowak L, Bregestovski P, Ascher P, Herbet A, Prochiantz A (1984). Magnesium gates glutamate-activated channels in mouse central neurons.. Nature.

[59] Bear MF, Kleinschmidt A, Gu QA, Singer W (1990). Disruption of experience-dependent synaptic modifications in striate cortex by infusion of an NMDA receptor antagonist.. J Neurosci.

[60] Bear MF (1996). A synaptic basis for memory storage in the cerebral cortex.. Proc Natl Acad Sci U S A.

[61] Bourne HR, Nicoll R (1993). Molecular machines integrate coincident synaptic signals.. Cell.

[62] Ramoa AS, Mower AF, Liao DS, Jafri SI (2001). Suppression of cortical NMDA receptor function prevents development of orientation selectivity in the primary visual cortex.. J Neurosci.

[63] Gandhi SP, Yanagawa Y, Stryker MP (2008). Delayed plasticity of inhibitory neurons in developing visual cortex.. Proc Natl Acad Sci U S A.

[64] Hensch TK, Stryker MP (2004). Columnar architecture sculpted by GABA circuits in developing cat visual cortex.. Science.

[65] Krug K, Akerman CJ, Thompson ID (2001). Responses of neurons in neonatal cortex and thalamus to patterned visual stimulation through the naturally closed lids.. J Neurophysiol.

[66] Durack JC, Katz LC (1996). Development of horizontal projections in layer 2/3 of ferret visual cortex.. Cerebral Cortex.

[67] Sengpiel F, Kind PC (2002). The role of activity in development of the visual system.. Curr Biol.

[68] Savage DD, Queen SA, Sanchez CF, Paxton LL, Mahoney JC (1992). Prenatal ethanol exposure during the last third of gestation in rat reduces hippocampal NMDA agonist binding site density in 45-day-old offspring.. Alcohol.

[69] Galindo R, Zamudio PA, Valenzuela CF (2005). Alcohol is a potent stimulant of immature neuronal networks: implications for fetal alcohol spectrum disorder.. J Neurochem.

[70] Constatinescu A, Diamond I, Gordon AS (1999). Ethanol-induced translocation of cAMP-dependent protein kinase to the nucleus.. J Biol Chem.

[71] Yang X, Horn K, Baraban JM, Wand GS (1988). Chronic ethanol adminstration decreases phosphorylation of cyclic AMP response element-binding protein in granule cells of rat cerebellum.. J Neurochem.

[72] Yang X, Horn K, Wand GS (1998). Chronic ethanol exposure impairs phosphorylation of CREB and CRE-binding activity in rat striatum.. Alcohol Clin Exp Res.

[73] Pandey SC, Roy A, Mittal N (2001). Effects of chronic ethanol intake and its withdrawal on the expression and phosphorylation of the CREB gene transcription factor in rat cortex.. J Pharmacol Exp Ther.

[74] Hsiao SH, Mahoney JC, West JR, Frye GD (1998). Development of GABAA receptors on medial septum/diagonal band (MS/DB) neurons after postnatal ethanol exposure.. Brain Res.

[75] Hsiao SH, Parrish AR, Nahm SS, Abbott LC, McCool BA (2002). Effects of early postnatal ethanol intubation on GABAergic synaptic proteins.. Brain Res Dev Brain Res.

[76] Issa NP, Trachtenberg JT, Chapman B, Zahs KR, Stryker MP (1999). The critical period for ocular dominance plasticity in the ferret's visual cortex.. J Neurosci.

[77] Kemeny V, Molnar S, Andrejkovics M, Makai A, Csiba L (2005). Acute and chronic effects of vinpocetine on cerebral hemodynamics and neuropsychological performance in multi-infarct patients.. J Clin Pharmacol.

[78] Malonek D, Dirnaql U, Lindauer U, Yamada K, Kanno I (1997). Vascular imprints of neuronal activity: relationships between the dynamics of cortical blood flow, oxygenation, and volume changes following sensory stimulation.. Proc Natl Acad Sci USA.

[79] Malonek D, Grinvald A (1996). Interactions between electrical activity and cortical microcirculation revealed by imaging spectroscopy: implications for functional brain mapping.. Science.

[80] Vanzetta I, Grinvald A (1999). Increased cortical oxidadtive metabolism due to sensory stimulation: implications for functional brain imaging.. Science.

[81] Thompson JK, Peterson MR, Freeman RD (2003). Single-neuron activity and tissue oxygenation in the cerebral cortex.. Science.

[82] Coppola DM, Purves HR, McCoy AN, Purves D (1998). The distribution of oriented contours in the real world.. Proc Natl Acad Sci U S A.

[83] Shapley R, Hawken M, Ringach DL (2003). Dynamics of orientation selectivity in the primary visual cortex and the importance of cortical inhibition.. Neuron.

[84] Somers DC, Nelson SB, Sur M (1995). An emergent model of orientation selectivity in cat visual cortical simple cells.. J Neurosci.

[85] Johnston MV, Jeon OH, Pevsner J, Blue ME, Naidu S (2001). Neurobiology of rett syndrome: a genetic disorder of synapse development.. Brain Dev.

[86] Johnston MV (2003). Brain plasticity in paediatric neurology.. Eur J Paediatr Neurol.

[87] Johnston MV (2004). Clinical disorders of brain plasticity.. Brain Dev.

[88] Siarey RJ, Villar AJ, Epstein CJ, Galdzicki Z (2005). Abnormal synaptic plasticity in the Ts1Cje segmental trisomy 16 mouse model of Down syndrome.. Neuropharmacology.

[89] Tully T, Bourtchouladze R, Scott R, Tallman J (2003). Targeting the CREB pathway for memory enhancers.. Nat Rev Drug Discov.

[90] Rose GM, Hopper A, De Vivo M, Tehim A (2005). Phosphodiesterase inhibitors for cognitive enhancement.. Curr Pharm Des.

[91] Krahe TE, Medina AE, Bittencourt-Navarrete RE, Colello RJ, Ramoa AS (2005). Protein synthesis independent plasticity mediates rapid and precise recovery of deprived eye responses.. Neuron.

[92] Haralick RM, Shanmugam K, Dinstein I (1973). Textural features for image classification.. IEEE Trans on system, man and cybernetics.

[93] Lowel S, Schmidt KE, Kim DS, Wolf F, Hoffsummer F (1998). The layout of orientation selectivity and ocular dominance domains in area 17 of strabismic cats.. Eur J Neurosci.

