# Supplementary material for: Phosphodiesterase Inhibition Increases CREB Phosphorylation and Restores Orientation Selectivity in a Model of Fetal Alcohol Spectrum Disorders
Source: PLoS One. 2009 Aug 14;4(8):e6643. doi: 10.1371/journal.pone.0006643 (PMC2721629; doi:10.1371/journal.pone.0006643)
Supplement: Results S1 — a)Effects of PDE type 1 inhibition on cAMP and pCREB levels. b)Alcohol exposure during the third trimester equivalent of human gestation does not affect visual acuity in the ferret. (0.04 MB DOC) [file pone.0006643.s001.doc]

*Krahe, Wang and Medina. PDE1 inhibition on Orientation selectivity*.

**Effects of PDE type 1 inhibition on cAMP and pCREB levels.**

In order to show that PDE inhibition can alter cAMP levels and CREB phosphorylation, 7 Long-Evans rats received 40mg/Kg of vinpocetine i.p. Animals were killed immediately after treatment or 1, 3, 6, 9, 16 and 24h thereafter. Cortices were collected and cAMP levels assessed by ELISA and CREB phosphorylation assessed by western blotting. We observed that, as early as 1h after vinpocetine injection, a 7% increase in cAMP was detectable. This modest increase in cAMP was accompanied by a 50% increase in pCREB levels. While cAMP levels tended to increase gradually up to a 30% increase at 9h, pCREB expression maintained a plateau from 1-16h after injection. After 24h both cAMP and pCREB returned to control levels (Figure S1). While this experiment was done in rats, it speaks favorably to our hypothesis that PDE inhibition affects cAMP and pCREB levels.

**Alcohol exposure during the third trimester equivalent of human gestation does not affect visual acuity in the ferret.**

Alcohol exposure during early and mid gestation can result on peripheral malformations such as microphthalmia and optic nerve hypoplasia [1] and abnormal curvature of the eyes [2,3]. However, it is unlikely that peripheral problems, especially optical problems, underlie the effects of alcohol on orientation selectivity described in the present manuscript. First, ethanol has been shown to result in a spectrum of ocular malformations only when administered to gastrulating embryos [4,5], a period that corresponds to the third week after fertilization in humans [4]. Second, we administered alcohol at a blood concentration of 250 mg/dl, less to the elevated blood alcohol concentration (430 mg/dl) required to elicit loss of optic nerve axons and decrease myelination [1,6]. Third, robust visual responses were preserved in alcohol-treated animals, as indicated by optical imaging of intrinsic signals and single-unit recordings.

To further test the hypothesis that the alcohol exposure protocol used here did not affect visual acuity, we quantified visual evoked responses (spikes per second) as a function of spatial frequency. To accomplish this, we first performed optical imaging of intrinsic signals (see methods) in an ethanol-treated and an untreated animal. After the orientation selectivity map was obtained, the glass cover slip and the agar were removed and an electrode was placed in the binocular region of V1 in an angle of approximately 45^o^. Single-units used in this study were separated by at least 100 µm along the electrode track. After the isolation of a single-unit, its receptive field was mapped and the optimal stimulus orientation, direction and velocity were determined qualitatively using a moving bar of light projected onto a tangent screen. Number of spikes per econd were then quantitatively determined for each cell by presenting computer generated moving gratings at seven different spatial frequencies (0.1, 0.15, 0.2, 0.25, 0.3, 0.4, and 0.5) in a pseudorandom sequence. Figure S2 shows that despite the striking differences observed in the orientation selectivity maps, both animals presented similar visual acuities.

Reference List

1. Stromland K, Pinazo-Duran M (2002) Ophthalmic involvement in the fetal alcohol syndrome: clinical and animal model studies. Alcohol Alcohol 37: 2-8.

2. Garber JM (1982) Steep corneal curvature in the fetal alcohol syndrome: a fetal alcohol syndrome landmark. J Am Optom Assoc 8: 641-644.

3. Miller M, Israel J, Cuttone J (1981) Fetal alcohol syndrome. J Pediatr Ophthalmol and Strab 18: 6-15.

4. Cook CS, Nowotny AZ, Sulik KK (1987) Fetal alcohol syndrome. Eye malformations in a mouse model. Arch Ophthalmol 105: 1576-1581.

5. Sulik KK, Johnston MC (1983) Sequence of developmental alterations following acute ethanol exposure in mice: craniofacial features of the fetal alcohol syndrome. Am J Anat 166: 257-269.

6. Harris SJ, Wilce P, Bedi KS (2000) Exposure of rats to a high but not low dose of ethanol during early postnatal life increases the rate of loss of optic nerve axons and decreases the rate of myelination. J Anat 197: 477-485.
