# Supplementary figures and images for: Phosphodiesterase Inhibition Increases CREB Phosphorylation and Restores Orientation Selectivity in a Model of Fetal Alcohol Spectrum Disorders
Source: PLoS One. 2009 Aug 14;4(8):e6643. doi: 10.1371/journal.pone.0006643 (PMC2721629; doi:10.1371/journal.pone.0006643)

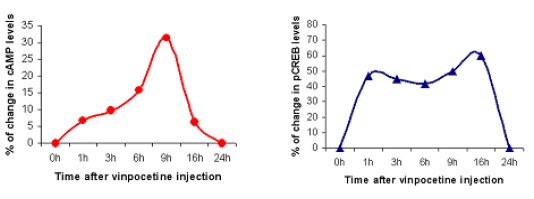

Supplement: Figure S1 — Changes in cAMP and CREB phosphorylation after vinpocetine. cAMP levels assessed by a commercially available immunoassay kit. CREB phosphorylation assessed by western blotting. Primary antibody: pCREB (Cell signaling, 1∶200 dilution). pCREB data was normalized by actin. (0.32 MB TIF) [file pone.0006643.s002.tif]

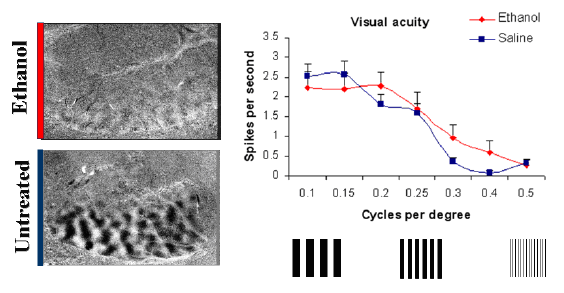

Supplement: Figure S2 — Number of spikes resulting from stimulation with different spatial frequencies. While the ethanol treated animal presented a severe impairment of the orientation selectivity map its response to changes in spatial frequency was similar to a control animal that exhibited a highly organized orientation selectivity map. (0.50 MB TIF) [file pone.0006643.s003.tif]
